# Geodiversity impacts plant community structure in a semi-arid region

**DOI:** 10.1038/s41598-021-94698-0

**Published:** 2021-07-27

**Authors:** Natalie De Falco, Reut Tal-Berger, Amgad Hjazin, Hezi Yizhaq, Ilan Stavi, Shimon Rachmilevitch

**Affiliations:** 1 The Jacob Blaustein Institutes for Desert Research, Ben-Gurion University of the Negev, 849900 Sede Boqer Campus, Israel; 2 French Associates Institute for Agriculture and Biotechnology of Drylands, The Jacob Blaustein Institutes for Desert Research, Ben-Gurion University of the Negev, 849900 Sede Boqer Campus, Israel; 3 The Albert Katz International School for Desert Studies, The Jacob Blaustein Institutes for Desert Research, Ben-Gurion University of the Negev, 849900 Sede Boqer Campus, Israel; 4 Department of Solar Energy and Environmental Physics, The Jacob Blaustein Institutes for Desert Research, Ben-Gurion University of the Negev, 849900 Sede Boqer Campus, Israel; 5 Dead Sea and Arava Science Center, 88820 Yotvata, Israel

**Keywords:** Biodiversity, Biogeography, Climate-change ecology

## Abstract

Geodiversity refers to the variety of geological and physical elements as well as to geomorphological processes of the earth surface. Heterogeneity of the physical environment has an impact on plant diversity. In recent years, the relations between geodiversity and biodiversity has gained attention in conservation biology, especially in the context of climate change. In this study, we assessed the spatial and temporal change in plant’s community structure in a semi-arid region, Sayeret Shaked Long Term Ecosystem Research (LTER) station, Israel. Vegetation surveys were conducted on different hillslopes, either with or without rock covers in order to study the spatial trends of hillslope geodiversity. The surveys were conducted for two consecutive years (2016 and 2017), of which the second year was drier and hotter and therefore permitted to investigate the temporal change of plant’s community structure. The results of the spatial trends show that (1) geodiversity increases vegetation biodiversity and promotes perennial plants and those of the temporal change show that (2) the positive effect of geodiversity on plants’ community structure and species richness is greater in the drier year than that in a wetter year. The main insight is that in these drylands, hillslopes with higher geodiversity appear to buffer the effect of drier years, and supported a more diverse plant community than lower geodiversity hillslopes.

## Introduction

Ecosystems are defined as complex communities of plant, animal and micro-organisms as well as the environments they are living and interacting with. The recognition and integration of the non-living features as part of the ecosystem is nowadays crucial^1^ even thus the abiotic world is often considered as function of the biotic one and not as its own^2^. An increase awareness on the relevance of the abiotic services led to the recognition in recent years of the interconnections between species, habitats and natural processes^3,4^. In fact, recently the term and concept of geodiversity was adopted in the scientific community to define the complex geomorphological features that contribute to ecosystem services^1,2,4,5^.

Geodiversity is defined as the natural assemblage of abiotic conditions within an ecosystem including its geological, geomorphological, and pedological features^6^. Geodiversity encompasses the substrates, landforms, and physical processes that govern habitat development and sustainability^5^. It can be addressed as variability between and among sites and it interested all Earth’s zones: litho-, hydro-, cryo- and atmo-sphere. The concept of geodiversity includes not only all its components but their relations and the link with the ecosystem they sustain^7^. In this framework, the vary geodiversity features play a crucial role in the conservation of habitats and landscape^1,8–10^.

An important component of geodiversity is the soil stoniness. The position of stones on the ground surface regulates hydrological process, as it affects infiltration and evaporation^11–13^. In fact, during rainfall, stones at the soil surface intercept raindrops^11^, reducing the splash formed by the impact force of raindrops on the soil^14^ with the consequences for processes of water overland flow and soil erosion^15,16^. On the other hand, stones increase water intake rates by preventing surface sealing and crusting^17^. Also, stones on the ground surface act as an isolation by reducing the soil temperature during the day and increasing the temperature during the night^18^. This temperature regulation resulted in effects on soil–water evaporation^19^. These processes are dependent on the stones’ size distribution and cover percentage^20^. Linked to the stoniness on the ground surface, the distribution of the stones throughout the soil profile is an important component of geodiversity^21–23^. The presence or absence of rock fragments affect the distribution of soil–water throughout the profile and the ramification of roots, and have an impact on soil temperature^24–26^. Characterization of soil profile is therefore crucial for understanding the complex impact of stoniness.

Heterogeneity of the physical environment, alongside with climatic variables, have a crucial effect on vegetation living conditions and biodiversity^5,27^. It was demonstrated that geodiversity affects the distribution of vegetation^28,29^, composition of soil microbes, and the resistance of plant to drought^30–32^. In drylands, understanding the relation between geodiversity-governed water distributions^21^ and plants viability is highly important. Recent studies from the semi-arid north-western Negev proposed that heterogeneous land units, dominated with partially-embedded stones in their ground surface, increase the spatial redistribution of water, with the resultant increase in water availability for shrubs^32^, improving their durability to prolong drought events^21^. Specifically, mass mortality of *Noaea mucronata* (Forssk.) shrubs was reported for hillslopes with low geodiversity level^21,32^. Recent studies proposed evidence on the buffering effect of geodiverse locations on vegetation diversity in a changing environment^33,34^ as geodiversity can improve adaptability of species and acts as microclimate refugia^35,36^. More empirical data from different climate zones are needed to support the geodiversity–biodiversity relations and to be implemented in conservation projects of natural ecosystem^6,37,38^.

The *N. mucronata* is a perennial shrub that dominates the north-western Negev (Fig. 2), which shows an adaptive response to dryland environments at numerous levels. The adaptation to water-limited environments shaped the plant’s metabolism and morphology. Aboveground adaptions comprise changes in leaf morphology, where the small narrow winter leaves that emerge after the rain (approximately in November) shed or turn into thorns during the dry season, reducing water loss through transpiration^39^. The seasonal difference in leaf morphology result in a total plant size variation from summer (smaller) to winter (larger). The green stems transform into grayish fissured bark, which enables the growth of young branches once the wet season starts^39^. The physiological traits of adaptions include the C_4_ carbon fixation pathway, which correlates with high efficiency in CO_2_ fixation and low transpiration loss under high temperature conditions^40^. Moreover, at the root level, *N. mucronata* adapt in association with ectomycorrhizal fungi, which improves the plants’ water and nutrient uptake^41,42^. The *N. mucronata* is considered as a landscape engineer^43–45^, which improves habitat conditions and facilitates herbaceous community in its surroundings by modifying microclimate (reduction of temperature stress due to the shading effect) and soil properties (increasing infiltration and reducing evapotranspiration)^43,46–48^. At the same time, it was reported that the *N. mucronata* may impose some negative effects, such as the suppression of annual plants^49^ through shading and competition^50^.

Our study investigates the spatial and temporal effect of geodiversity on plant community structure and on our model species *N.mucronata*. The importance of our results is its link with the climatically conditions and the novelty of the semi-arid region case. The study objectives were to assess during two compared year, a wetter and a drier one, the effect of geodiversity features such as stoniness and depth of the soil profile (1) on the physiological conditions of *N. mucronata* shrubs, and (2) on plant’s community structure and diversity. Our overall hypotheses are that an increase of geodiversity will (1) improve the physiological conditions of *N. mucronata* shrubs and (2) enhance plant diversity﻿.

## Materials and methods

### Regional settings

**Figure 1:**
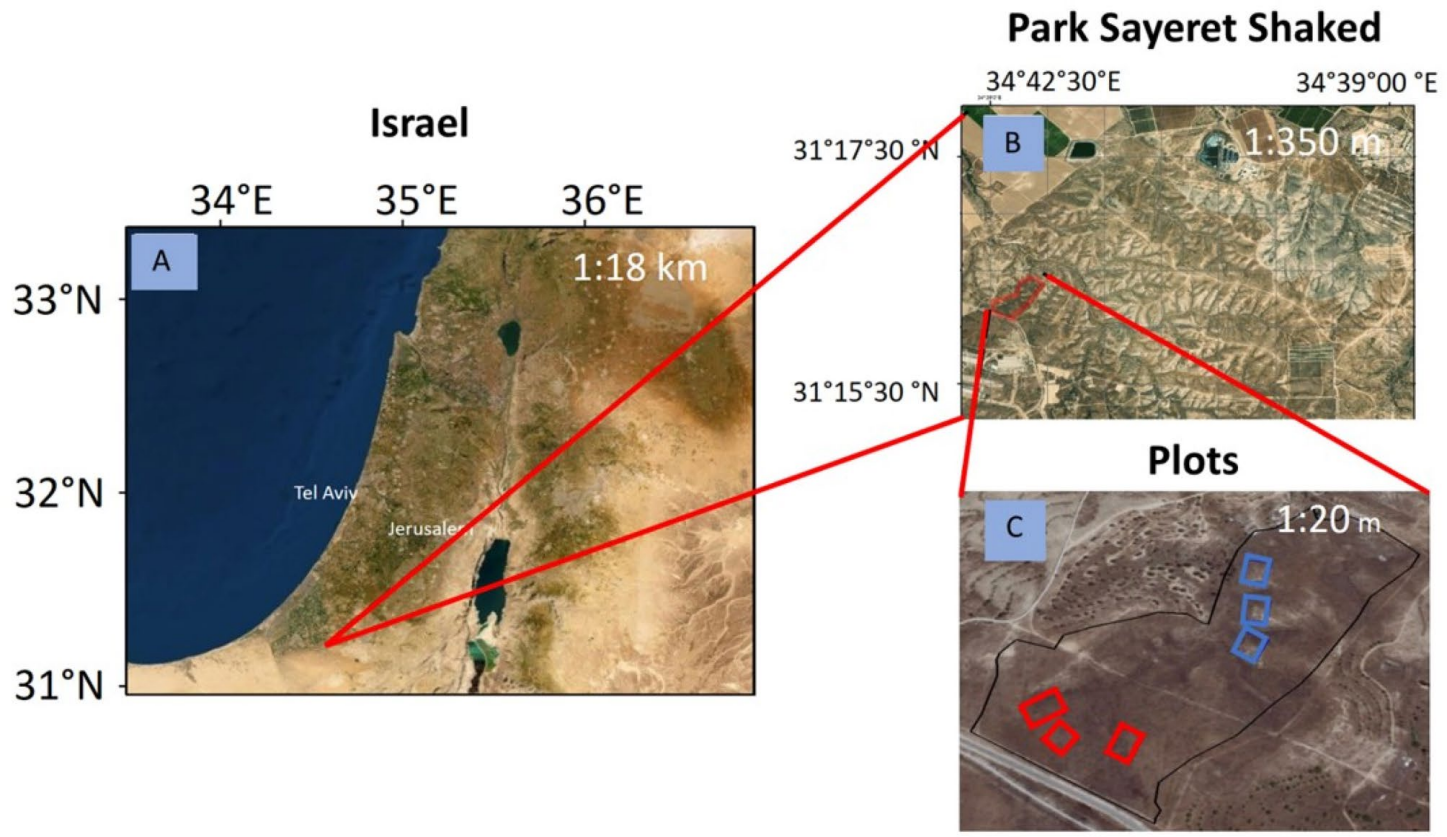
Maps of the study area. Sayeret Shaked LTER location in Israel (**A**), the location area (**B**) and the plots (**C**) where the red squares are the homogeneous plots and the blue squares the heteregenous ones (Google Earth). Maps data, Google 2021, CNES/Airbus, Maxar Tecnologliges, satellite image accesed through Google Earth.

**Figure 2:**
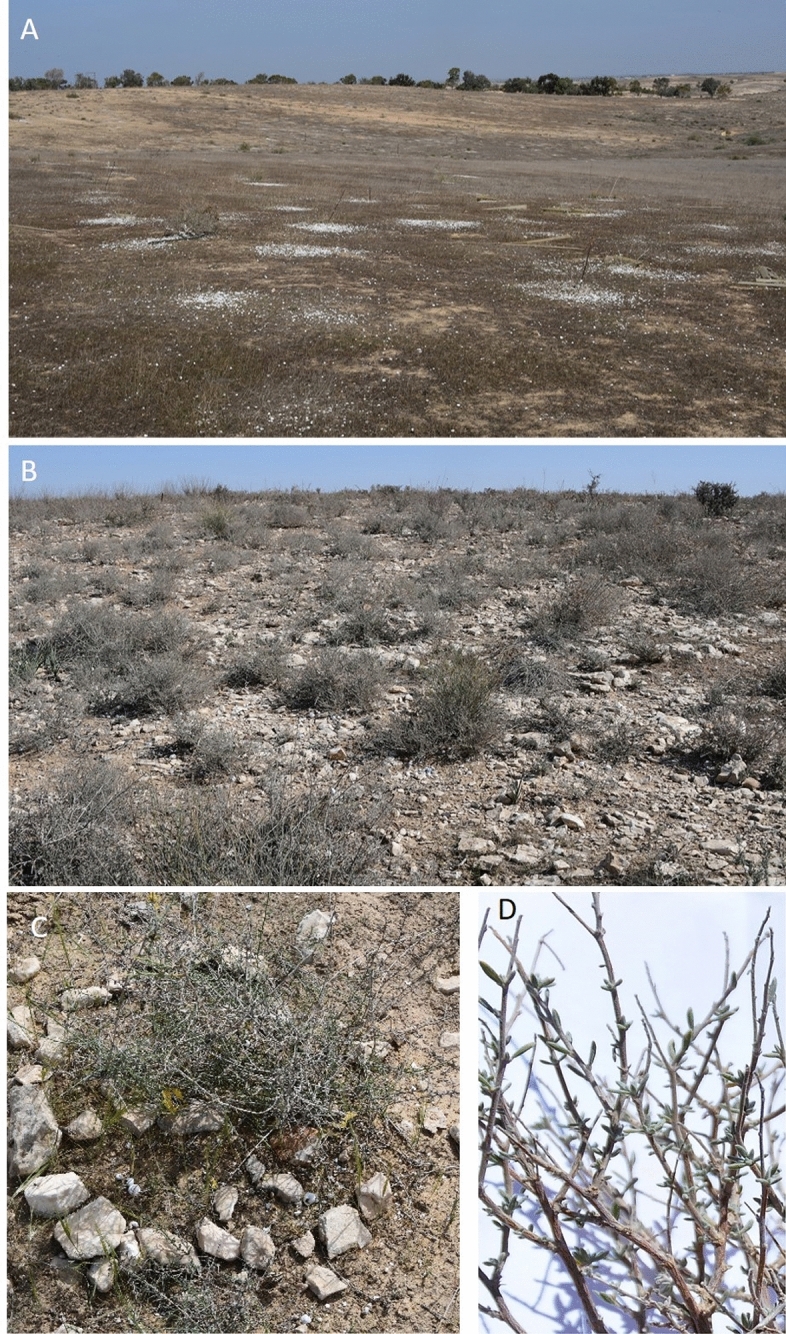
A view of a homogeneous (**A**) and a heterogeneous (**B**) hillslopes. In the homogeneous hillslopes, the white spots are piles of snails in the vicinity of dead shrubs. The piles indicate the location of dead shrubs. At the same time, most of the shrubs in the heterogeneous hillslope are alive, and survived the prolonged drought of 2008–2009. *N. mucronata* during spring season (**C**). A detail of winter leaves (**D**). Photos courtesy of Prof. Hezi Yizhaq.

The study was conducted between February 2016 and May 2017 in the fenced area (no access for livestocks since 1990s) of the Sayeret Shaked Park (~ 20 ha) (Fig. 1) with the permission of the Israel’s LTER Network. The park is located in the semi-arid north-western Negev of Israel (31° 17′ N, 34° 37′ E; 200 m.a.s.l.), where annual average rainfall is ~ 165 and standard deviation of 58 mm/year^21^. The site’s soil is aeolian loess, with sandy loamy to loamy sand texture^51^. The park is covered with dwarf shrubs (0.1–0.6 m tall) such as *Atractylis serratuloides* Cass., *Thymelaea hirsuta* (L.) Endl., and the dominant *N. mucronata* shrubs. The site has a large number of annuals, geophyte (e.g., *Asphodelus ramosus* L.) and hemicryptophyte species, as well as a rich community of biological crusts^50,52–55^. In a previous study, a geomorphological survey followed by hillslopes mapping took place in Sayeret Shaked starting in July 2015^21^. The results showed the presence of either hillslopes without rock fragment cover and hillslopes with substantial (~ 30%) rock fragment cover (Fig. 2). Following the study, three low-geodiversity hillslopes, with a thick (> 1 m) and non-stony soil layer, without rock fragment cover or rock fragment content in the soil profile (homogeneous: HM; Fig. 2A), and three high-geodiversity hillslopes, with a thin (~ 0.1 m) stony soil layer, rock fragment cover (~ 30%) and rockfragment content in the soil profile (~ 35%) (heterogeneous: HT; Fig. 2B) were selected for the study. The mean (± SE) slope incline of all the hillslope is 5° (±)^22^. Distance between two adjacent hillslopes was at least 100 m. On each of these hillslopes, a 400 m^2^ (20 × 20 m) plot was established for data collection.

To study the effect of the geodiversity features on plant community, we assessed the plant diversity at a once-a-month frequency over the growing season (Feb–May/June) of two sequential years (2016–2017). In each of these years, the last cycle of data collection took place when the annual plants were dried out, and at the point where the *N. mucronata* ‘s ‘winter leaves’^56^ have disappeared. Data of precipitation, solar radiation, soil temperature and soil moisture at different depths (25 and 50 cm) were obtained from the meteorological station located in the LTER site.

### Vegetation survey

The *N. mucronata* was the only perennial plant present in both HT and HM hillslope, making it the best-fit model species to assess the year-round differences in plant viability. In each plot (n = 6) we studied three individual shrubs, to a total of nine individuals per hillslopes type. In order to monitor the shrub’s morphological changes during the year, we measured the maximum length of green branches. Also, we measured the plant's size by measuring the maximum length from a green part to another on a north–south and east–west axes, as well as the shrub height. We multiplied the three axes to calculate the maximum plant size. In addition to these nine *N. mucronata* plants, we sampled leaves (winter leaves only) from other nine randomly selected *N. mucronata* plants to estimate their physiological condition, through measuring the relative water content (RWC), membrane stability (EC), carbon–nitrogen ratio (C:N), and chlorophyll content.

### Biochemical analysis

#### Relative water content (RWC)

We added 3–5 g of *N. mucronata* young leaves of each individual to a 50 ml vial with a wet tissue to maintain humidity. The leaves were weighted for fresh weight using Sartorius AG Göttingen CP225D, Germany. The samples were submerged in de-ionized water for 24 h and then weighted for turgor weight. Additionally, the samples were dried at 65 °C for 24 h in the oven for dry weight.

#### C:N ratio

Few leaves from each shrub of *N. mucronata* were collected for total organic carbon (C_org_) and total nitrogen content (N_tot_) analysis, dried at 65 °C for 12 h and manually ground by mortar and pistil. Of these samples, 20 mg were put in a C–N analyzer (CHNS Elemental Analyzer, Thermo Scientific, USA).

#### Membrane stability index (MSI)

From each *N. mucronata*, 20–30 leaves were collected and placed in 50 ml vials filled with 20 ml of double distilled water (DDW). The electrolyte’s electric conductivity (EC) was measured with a probe (Eutech Instruments, CON 510, Singapore), as initial leakage (C_i_). The samples were then placed on a laboratory shaker (TOS-4838PD, MRC Lab Instruments, Holon, Israel) for 12 h at 200 rpm and the EC was measured again as C_*r*_. The samples were then autoclaved to blast cell membrane, and the maximum conductivity (C_m_) was measured. Membrane stability index (MSI) was calculated according to Eq. (1):

1 $$MSI= 100* \frac{{C}_{f}{-C}_{i}}{{C}_{m}- {C}_{i}}$$

#### Chlorophyll content

10 leaves of *N. mucronata* were collected and add into a 2 ml Eppendorf with 1 ml of Dimethyl sulfoxide (DMSO), covered with aluminum foils. In the laboratory, vials were kept in a 65 °C incubator for 72 h and then centrifuged for 14,000 rpm at 20 °C for 10 min. 200 μl of the supernatant was added to a 96 wells plate and the absorption was read with Epoch™ spectrophotometer (BioTek Instruments, Inc., Vermont, USA). Chlorophyll *a* (*Chl a*), contents was calculated using Eq. (2)^57^:

2 $$Chl a= 13.34*{A}_{666}-4.85*{A}_{650}$$

### Vascular plant diversity

To measure plant diversity, we identified all the vascular plants to the species level along 3-m transects (one transect per plot), counted the number of individuals per species, and recorded the total cover per species. The monitoring of transects was conducted at a once-a-month frequency throughout the growing season (Feb–June 2016 and Feb–May 2017: a total of 11 sampling cycles). Due to overlapping plants, vegetation’s total cover could exceed 100%. We classified all plant species into three life forms: annual, perennial and herbaceous perennial. The use of any plants in this study was in accord with national guidelines. The formal identification of the plant material was performed by Prof. Rachmilevitch. We did not use voucher specimen.

In order to determine the plant diversity in the different hillslope, we calculate species richness (n) as the number of species present at the site, and species abundance as the total number of plants present at the site (N). In order to determine the diversity in the communities, we calculate the Shannon Diversity Index (Eq. 3) and Shannon Evenness Index (Eq. 4) and the Simpson Index (Eq. 5),^58,59^

3 $$Shannon\, Diversity\, Index \left(H\right)=-\sum\limits _{i=l}^{s}{p}_{i}\;\mathrm{ln}{(p}_{i})$$

4 $$Shannon \,Evennes\, Index \left(SEI\right)=-\sum\limits _{i}^{s}\frac{{p}_{i}\;\mathrm{ln }({p}_{i})}{\mathrm{ln}(s)}$$

5 $$Simpson\, Index \left(D\right)=\frac{1}{\sum\nolimits _{i=l}^{s}\;{p}_{i}^{2}}$$

where p is the proportion (n/N) of individuals of one particular species found (n) divided by the total number of individuals found (N), ln is the natural log, Σ is the sum of the calculations, and s is the number of species.

### Data analysis

Nonparametric Mann–Whitney U test was performed to analyze the meteorological data (averaged solar radiation, total rain precipitation, averaged soil temperature and soil moisture at 25 and 50 cm depth) of the LTER station of year 2016 and 2017. Kruskal–Wallis one-way analysis of variance was used for averaged data of the different hillslopes (HM vs HT) of biochemical data (RWC, MSI and chlorophyll *a* content) as well as for estimated size of shrubs. Non-parametric Dunn's test for pairwise multiple comparisons was used to compared biochemical data of 2016 vs 2017 in the different hillslopes. Analysis of similarity (ANOSIM) was performed to explain plant diversity in the different hillslopes per hillslope and per year. Life form composition was analyzed with Pearson Chi-Square. All statistical were conducted using SPSS Statistics (SPSS, IBM Corp, Version 26.0. Armonk, NY, U.S.A.). Analysis of similarities was carried out in R^60^.

## Results

**Figure 3:**
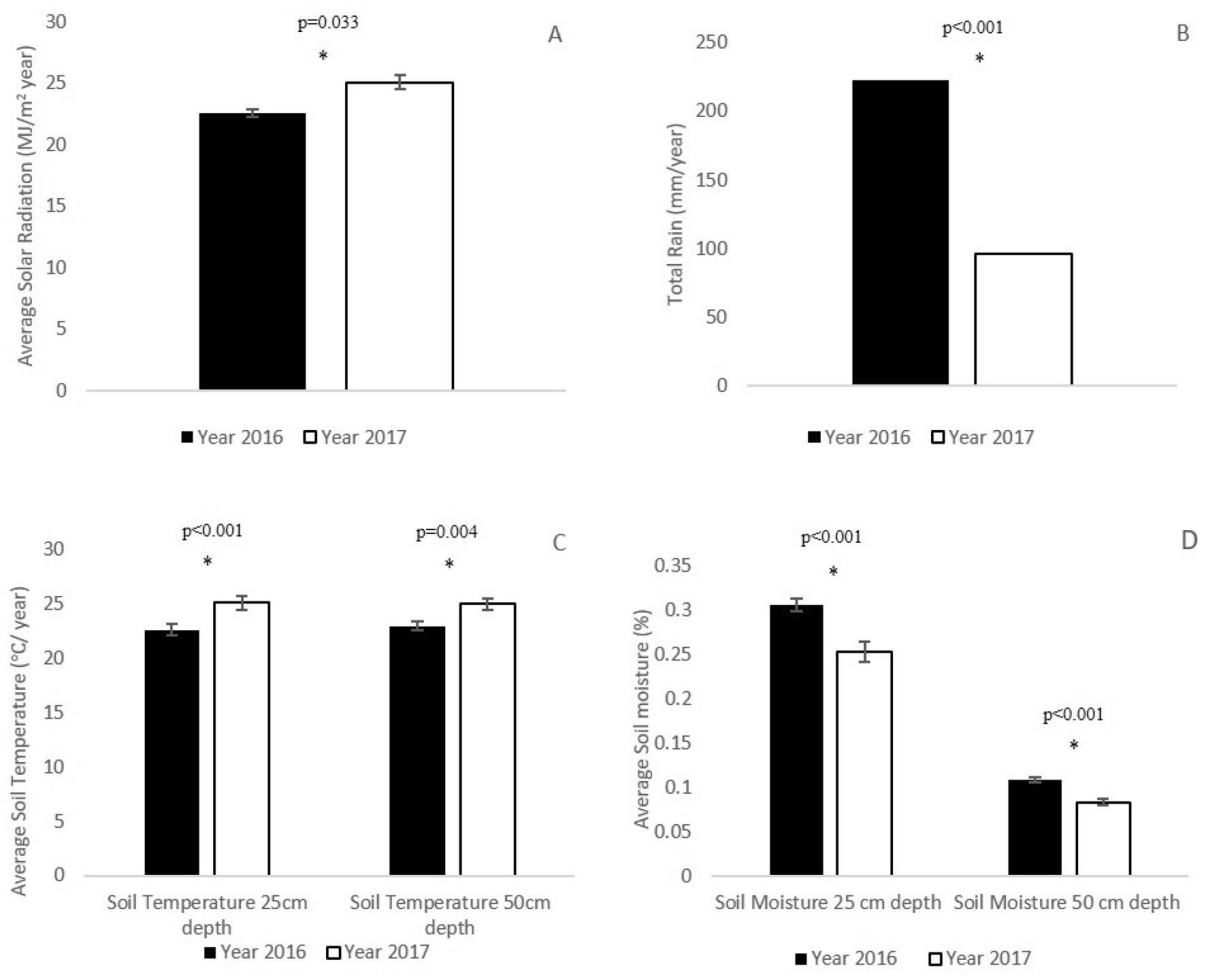
Average solar radiation (± SE) (**A**), total rain (**B**), average soil temperature (± SE) (**C**), and average soil moisture (± SE) (**D**), for 2016 (n = 269) and 2017 (n = 337). For relative trend of meteorological parameters in the period 2000–2015 see SI 1.

The two years of sampling were characterized by two different rain regimes (Fig. 3B), where 2016 was substantially wetter compared to 2017 (223 and 96 mm, respectively: Fig. 3B, Mann–Whitney Ranks Sum Test *p* < 0.001). Data of total solar radiation (Fig. 3A) indicates too a significant difference between 2016 and 2017 (22.6 and 25.1 MJ/m^2^ year of Solar Radiation respectively, Mann–Whitney Ranks Sum Test *p* = 0.033). This resulted in increase of soil temperature in 2017 at both 25 and 50 cm soil depths (Fig. 3C), as well as the reduction of soil moisture at both depths (Fig. 3D).

We did not find significant differences in physiological measurements of *N. mucronata* plants between the two hillslope types throughout the two years of sampling (SI 2). For the phenological measurements, the estimated mean *N. mucronata* size was greater in HM hillslopes than that in HT hillslopes (Kruskal–Wallis one-way analysis of variance, *p* < 0.0001).

**Figure 4:**
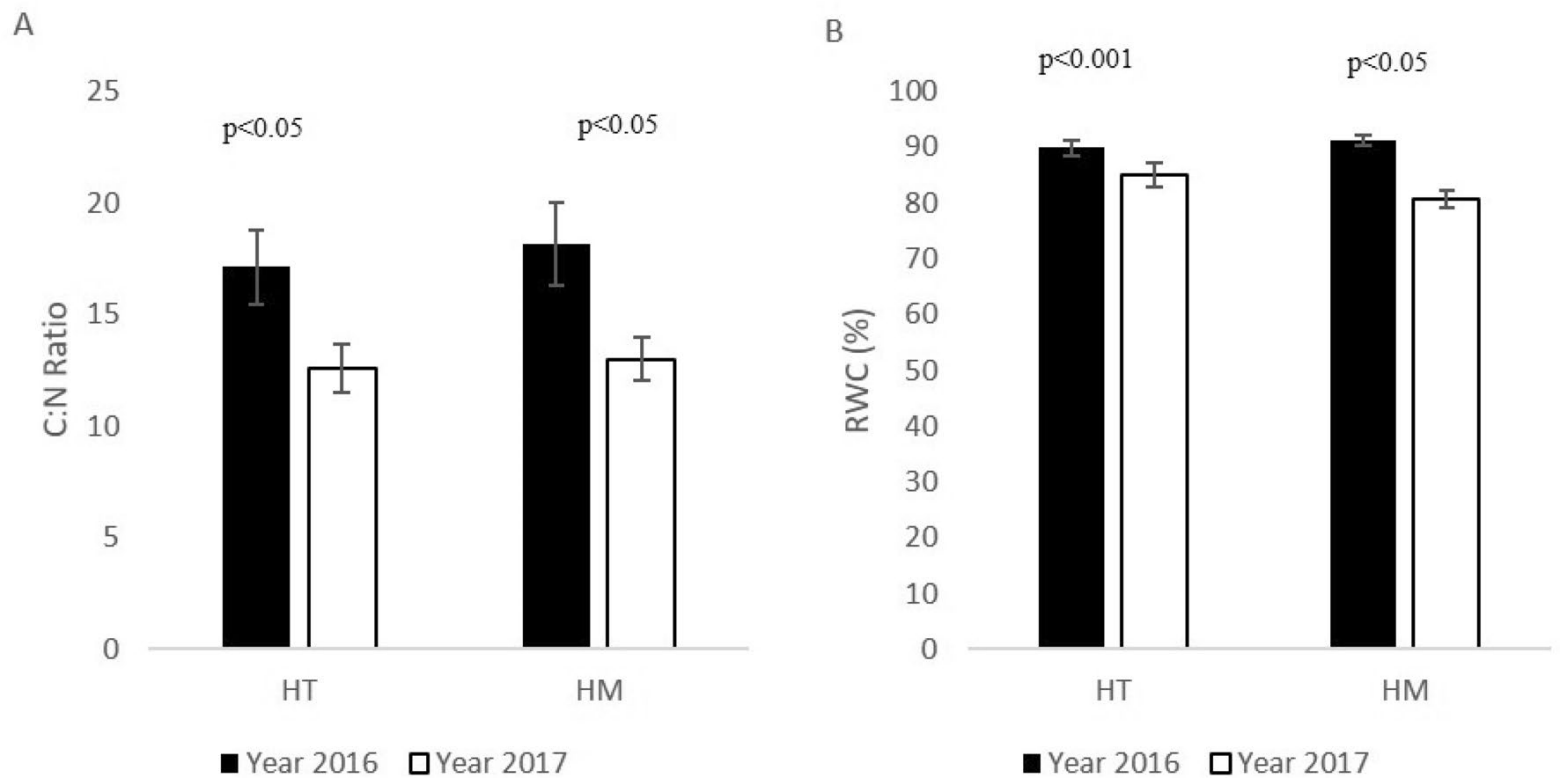
Mean C:N ratio (**A**) and Relative Water Content (RWC: **B**) in *N. mucronata* leaves at the heterogeneous and homogeneous hillslopes, for 2016 and 2017.

Nevertheless, once we plotted the data for different years (2016 vs 2017), we found a significant difference for most of the parameters between the two years, and especially regarding the C:N ratio and RWC. In 2016, *N. mucronata* had a higher C:N ratio (HT, Dunn’s Method: *p* < 0.05, HM Dunn’s Method: *p* < 0.05, Fig. 4A) and higher RWC (HT Holm–Sidak method: *p* < 0.001, HM Dunn’s Method: *p* < 0.05, Fig. 4B) than these in 2017. For the C:N ratio, this trend was similar for HT and HM hillslopes (a decrease of 26% and 28% from 2016 to 2017 in HT and HM, respectively). At the same time, the decrease in RWC was much smaller in HT hillslopes (5%) than that in HM hillslopes (11%).

### Plant diversity

**Figure 5:**
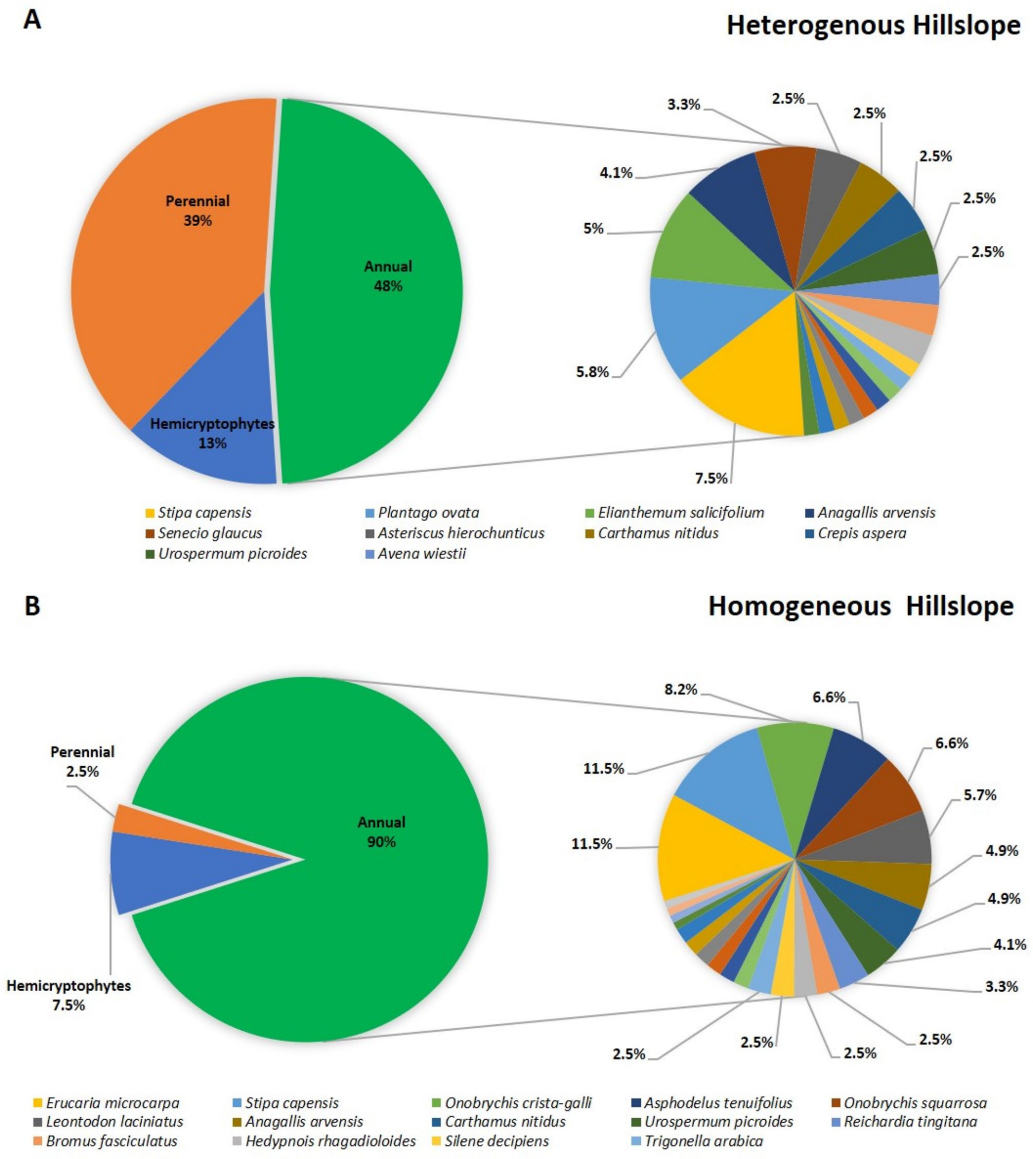
Life form composition in heterogeneous (**A**) and homogeneous (**B**) hillslopes. Differentiation among perennial (red), herbaceous perennial (blue), and annuals (green) is presented in the left circles, and species-level differentiation of annuals is presented in the right circles (only species that represent more than 2% were included in the figure). For the total list of the species SI 3–4.

We found that plant diversity can be explained by hillslope type (Analysis of similarity: R = 0.36, *p* = 0.0001) (Fig. 5A,B). In addition, we measured the effect of sampling year (SI 6 A, B, C and D), which was also significant, though the value explained was lower than the hillslope type (R = 0.11, *p* = 0.002). Moreover, we found a significant difference in life form composition, considering all species found, between the hillslopes (Pearson chi-square: Chi Square: 56.7, df = 2, *p* < 0.0001). HT plots had a higher mean value of perennial (39% in HT compared to 3% in HM) as well as higher mean value of herbaceous perennial (13% in HT compared to 7.5% in HM) (Fig. 5A,B). Specifically, in HM we found two shrubby species (*Pituranthos tortnousus* and *N. mucronata*), and a total of 3 individuals, while in the HT we found six shrubby species (*N. mucronata, A. articulata, Dianthus monadelphus subsp. judaicus, Salvia lanigera* Poir.*, Helianthemum stipulatum* (Forssk.) C. Chr. and *Helianthemum lippii* (L.) Dum. Courset) and a total of 47 individuals. At the same time, the mean value of annuals was significantly higher in HM (90% in HM compared to 48% in HT). The annual plant community in HM encompassed of 25 species and 110 individuals, while in the HT it encompassed 20 species and 58 individuals.

The accumulated cover of four species contributed to 52.8% of the differences between the two hillslope types. These species included *Stipa capensis* Thunb. (19.6%), *N. mucronata* (18.8%), *Anabasis articulata* (Forssk.) Moq. (8.6%), and *Onobrychista crista-gali (L.) Lam.* (5.8%) (Similarity percentage analysis, SI 5). The main differences were caused by two perennial plants that were absent from the HM transects: *N. mucronata* and *A. articulata*. Additionally, once we plotted the annuals’ diversity data per year (SI 6 A–D), we found that the annual community structure in the HT hillslopes during the shift from 2016 to 2017 became more even, with a more uniform abundance of species (SI 6 A and B). An opposite trend was found for the HM hillslopes, where an increase in abundance of dominant species was observed (SI 6 C and D).

Species richness and abundance are reported in Table 1. Species richness was similar in HT and HM hillslopes (31 and 30 species, respectively). Once comparing 2016 to 2017 year—with equal species richness in both of the hillslope types but different structures and abundance in 2016—HT hillslopes faced an increase in 2017, while HM hillslopes faced a decrease﻿.

**Table 1:** Mean species richness and species abundance in the Heterogeneous (HT) and homogeneous (HM) hillslopes in 2016 and 2017 years.

Plot	Species richness (n)	Species abundance (N)	Shannon Diversity’s Index (H)	Simpson Diversity’s Index (D)	Shannon Evenness Index (E)
HT (2016–2017)	31	122	1.71 ± 0.01	2.59 ± 0.01	0.50
HM (2016–2017)	30	121	1.65 ± 0.01	2.59 ± 0.01	0.49
HT (2016)	19	141	1.43 ± 0.02	2.23 ± 0.02	0.49
HT (2017)	22	102	2.02 ± 0.02	4.01 ± 0.01	0.65
HM (2016)	19	71	1.83 ± 0.02	3.78 ± 0.01	0.62
HM (2017)	17	51	0.90 ± 0.01	1.47 ± 0.04	0.32

To gain more insights about the community structure, we calculated the Shannon Diversity Index and Simpson Diversity Index (Table 1). Overall, HT hillslope had a higher diversity (Shannon Diversity Index in HT was 3.5% higher than that in HM). Further, once we calculated the yearly data, we found that in 2017 the drop of diversity in HM hillslopes was significant, whilst a significant increase was observed for HT hillslopes. Moreover, in 2017, the Shannon Evenness Index showed an increase in HT hillslopes and a decrease in HM hillslopes. This suggests that in HM hillslopes, the functional group encompass few dominant species with high abundance and few sparser species with low abundance. In HT hillslopes, the community structure was more even.

## Discussion

The aims of our study were to assess the spatial effect of geodiversity, express as stoniness and depth of the soil profile in different hillslopes, on the physiological conditions of *N. mucronata* shrubs and plant’s community structure and diversity. We showed that the geodiversity features promote a more diverse plant community (Fig. 5A,B). During the two years monitoring (2016–2017) the second year happened to be significant drier with repercussion on soil temperature and moisture at different depth (Fig. 3). This fact allowed us to have insights about the temporal effect of geodiversity features to mitigate drier years. In fact, annual communities on hillslopes with lower geodiversity tend to suffer a decrease in diversity during the drier year (SI 6 and Table 1). Previous studies from the semi-arid north-western Negev suggested that hillslope-scale geodiversity improves the source–sink relations^29^ and positively affect soil quality and geo-ecosystem functions. Specifically, it was reported that the HT hillslopes had 22% greater mean hygroscopic moisture^22^ then that in HM hillslopes. This result can indirectly indicates a similar availability of soil water for plants. The comparatively favorable conditions in the HT hillslopes were proposed to be the key of survival of *N. mucronata* plants during the mass mortality that occurred during different drought events started in 1999^32^.

Geodiversity components such as geomorphology, topography, geology, and hydrology are associated with energy and nutrients, which regulate biodiversity^61,62^. Nevertheless, only recently, the impact of geodiversity on biodiversity has gained attention^63–66^. Considering global climatic change, it was proposed that high-geodiversity land units are potentially more capable to support biodiversity because of their intrinsic resilience^65,67–69^. In our study, the favorable soil conditions in HT hillslopes were not straightforward translated into a better physiological state of plants (SI 2), as we expected in our first hypothesis. Our biochemical analysis did not show a prominent effect of geodiversity features over the physiological conditions of *N.mucronata*, when we compared the plots (HT vs HM) (SI 2). Despite that, during 2017, the shrubs in HM hillslopes faced stronger water stress compared to these in HT hillslopes (Fig. 4B). The positive effect of increased geodiversity on hygroscopic moisture^22^, and the overall higher soil–water content in HT hillslopes^70^, might explain the reduced water stress for shrubs in HT hillslopes^32,71^.

The shrub patch is the driver of the cyclic succession of plants community^48,72–74^. According to the biodiversity cyclic hypothesis^48^, once the patch is consolidated, the dissimilarity in community structure between the shrubby patches and interpatch spaces increases. Therefore, shrublands patchiness is critical for sustaining spatial heterogeneity^75^. In our study region, the HT hillslopes have higher patchiness, mainly due to higher geodiversity that supports shrub durability to droughts. Regarding our second objective on the effect of geodiversity on the plant community structure, we found that the perennials in HM were 2% of the community structure, whilst being 38% in HT (Fig. 5A,B). In HM, the annuals contributed 90% of the total community structure, while in HT they contributed 48% (Fig. 5A,B). The high cover of annuals in the HM hillslopes compared to the HT hillslope displays one possible response of the ecosystem to prolonged droughts that is a transition from shrubland to grassland. This transition did not occur in the HT hillslopes due to the effect of geodiversity that increase their resistance to drought. The ecosystem in the HM does not collapse to the bare soil as predicted by many mathematical models of vegetation patterns in drylands that include only wood vegetation but it is actually shifted to another stable state^76,77^.

The annual community structure faced a shift from the wetter 2016 to the dryer 2017 in both hillslopes (SI 6 A–D). In HT hillslopes, the annuals community become more even, having fewer dominant species with high abundance (SI 6 A and B). At the same time, in HM hillslopes, the annuals’ community structure became less even (SI 6 C and D). In our diversity analysis, we showed that in the drier year, plant diversity increased in HT hillslopes (Table 1) and decreased in HM hillslopes. Different simulation studies, where plant communities underwent abiotic stresses related to climate changes—such as higher temperature or lower precipitations—showed similar results^78,79^. It was shown that community response to changes is rapid (two seasons), and that stresses can change patterns of plant dominance and evenness^78,79^. Changes in dominance in community composition can have consequences for species coexistence and ecosystem functions^80^. Recent studies show that locations with high geodiversity have the potential role to act as hotspot for biodiversity and to maintain community’s structure due to their function to buffer climatic changes^66,81^. In our study, we show that the effect of geodiversity features on community structure and species richness is greater in the drier year than that in a wetter year. Our results suggested that hillslopes with higher geodiversity might be in situ refugia that facilitate the existing community and in shifting climate periods might serve as biodiversity spots^82–86^.

Our results aligned with the findings of recent studies that suggest that integrating geodiversity in conservation nature projects becomes a necessity to protect ecosystems and their services^10,65,87^ and offer the opportunity to broad the data availability. In fact the connection between biodiversity and geodiversity allow to use geodiversity measurements, more spatial consistent and therefore available at broader scale, and relating them to biodiversity^64,88^.

## Conclusion

The motivation of this study was to understand how different geodiversity features, expressed by the degree of stoniness and soil thickness, affect the physiological state of *N. mucronata* and the plant life form variability. In conclusion, our data show that in a semi-arid regions, hillslopes with higher geodiversity better buffer the effect of drier years, and supported a more diverse plant community compared to lower geodiversity hillslopes. The potential role of sites with higher geodiversity to mitigate climate change effects in terms of persistence of biodiversity should be take into consideration when planning for conservation actions and ecosystem management^84,89,90^ In this context, additional studies should be conducted in other drylands of the world in order to verify the mechanisms through which geodiversity regulates the structure and composition of vegetation community.

## Supplementary Information

Supplementary Information.
