# Supplementary material for: Geodiversity impacts plant community structure in a semi-arid region
Source: Sci Rep. 2021 Jul 27;11:15259. doi: 10.1038/s41598-021-94698-0 (PMC8316420; doi:10.1038/s41598-021-94698-0)

SI 1. Quantitative results of different weather paramenters from the LTER Station in Sayeret Shaked. Each point represent the average of the year (expected for the Total Rain) ± SE from 2000 till 2015. The sample size is respectively 2000 n=366, 2001 n=365, 2002 n=364, 2003 n=365, 2004 n=365, 2005 n=324, 2006 n=356, 2007 n=288, 2008 n=366, 2012 n=365, 2013 n=254, 2014 n=260, 2015 n=365.

Years 2009-2011 are omitted due the missing data (n=94, n=50 and n=240 respectively).

*
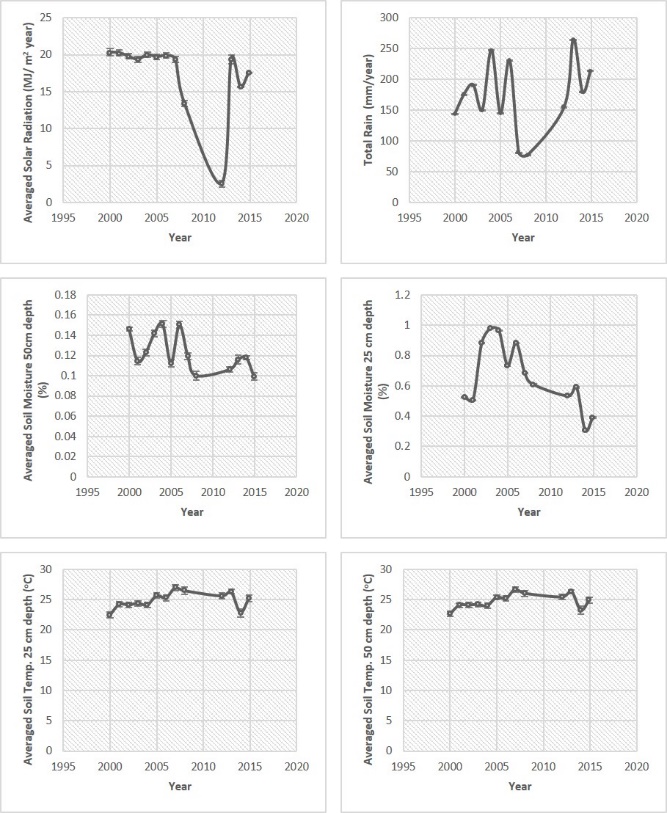
*

SI 2. Mean ± SE of all biochemical and phenological measurements takes from *N. mucronata* in heterogeneous (HT) and homogeneous (HM) hillslopes for the years 2016-2017. The *p* value referred to Kruskal–Wallis one-way analysis of variance test.

| Measurement | HT | | HM | p value |
| --- | --- | --- | --- | --- |
| Relative water content (%) | | 90.4 ± 2.9 | 89.03 ± 4.7 | >0.05 |
| Membrane stability (%) | | 69.4 ± 7.3 | 74.0 ± 5.6 | >0.05 |
| Chlorophyll a content (µg/mg) | | 0.063 ± 0.002 | 0.059 ± 0.002 | >0.05 |
| C:N Ratio | | 15.2 ± 1.1 | 16 ± 1.2 | >0.05 |
| Estimated size (m^3^) | | 0.14 ± 0.02 | 0.42 ± 0.02 | <0.0001 |

SI 3. List of the annual species and their percentage cover in the homogeneous hillslope.

| Annual  Homogeneous Hillslope | Total Cover (%) |
| --- | --- |
| Erucaria microcarpa | 11.5 |
| Stipa capensis | 11.5 |
| Onobrychis crista-galli | 8.2 |
| Asphodelus tenuifolius | 6.6 |
| Onobrychis squarrosa | 6.6 |
| Leontodon laciniatus | 5.7 |
| Anagallis arvensis | 4.9 |
| Carthamus nitidus | 4.9 |
| Urospermum picroides | 4.1 |
| Reichardia tingitana | 3.3 |
| Bromus fasciculatus | 2.5 |
| Hedypnois rhagadioloides | 2.5 |
| Silene decipiens | 2.5 |
| Trigonella arabica | 2.5 |
| Astragalus asterias | 1.6 |
| Avena sterilis | 1.6 |
| Calendula arvensis | 1.6 |
| Centaurea pallescens | 1.6 |
| Helianthemum salicifolium | 1.6 |
| Hippocrepis multisiliquosa | 1.6 |
| Aegilops triuncialis | 0.8 |
| Avena wiestii | 0.8 |
| Senecio glaucus | 0.8 |
| Silene alexandrina | 0.8 |

SI 4. List of the annual species and their percentage cover in the heterogenous hillslope.

| Annual  Heterogenous Hillslope | Total Cover (%) |
| --- | --- |
| Stipa capensis | 7.4 |
| Plantago ovata | 5.8 |
| Elianthemum salicifolium | 5.0 |
| Anagallis arvensis | 4.1 |
| Senecio glaucus | 3.3 |
| Asteriscus hierochunticus | 2.5 |
| Carthamus nitidus | 2.5 |
| Crepis aspera | 2.5 |
| Urospermum picroides | 2.5 |
| Avena wiestii | 1.7 |
| Erucaria microcarpa | 1.7 |
| Pteranthus dichotomus | 1.7 |
| Astragalus asterias | 0.8 |
| Hordeum spontaneum | 0.8 |
| Leontodon laciniatus | 0.8 |
| Malva aegyptia | 0.8 |
| Phagnalon rupestre | 0.8 |
| Phagnalon rupestre | 0.8 |
| Pterocephalus plumosus | 0.8 |
| Reichardia tingitana | 0.8 |
| Sonchus oleraceus | 0.8 |

SI 5. Hillslope type effect on plant adaptability for the species that their total-cover explains 55.9% of the difference between heterogeneous and homogeneous hillslopes

| Species | Life form | Contrib.% | Cumulative % |
| --- | --- | --- | --- |
| S. capensis | Annual | 19.6 | 19.6 |
| N. mucronata | Perennial | 18.8 | 38.4 |
| A. articulata | Perennial | 8.6 | 47 |
| O. gali | Hemicryptophyte | 5.8 | 52.8 |

SI 6. Annual community structure in the heterogeneous hillslopes in 2016 (A) and 2017 (B), and in the homogeneous hillslopes in 2016 (C) and 2017 (D).


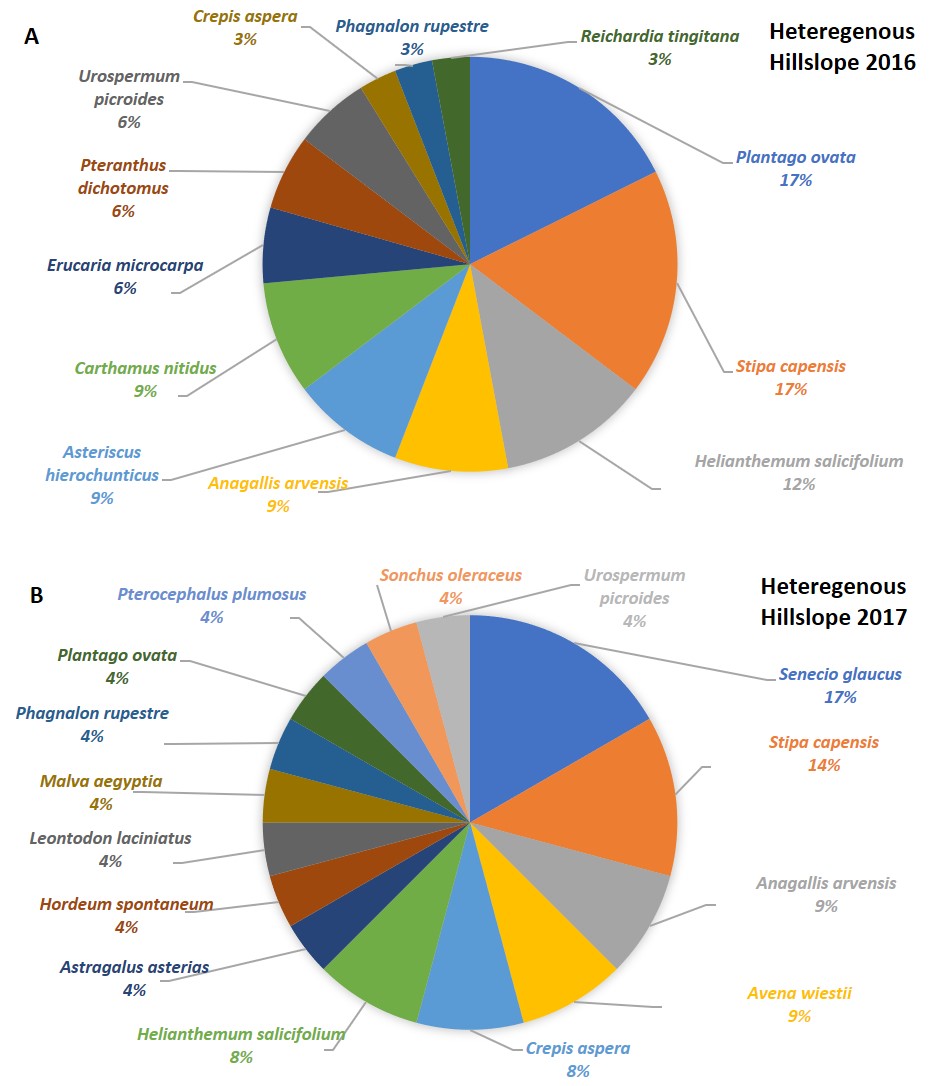


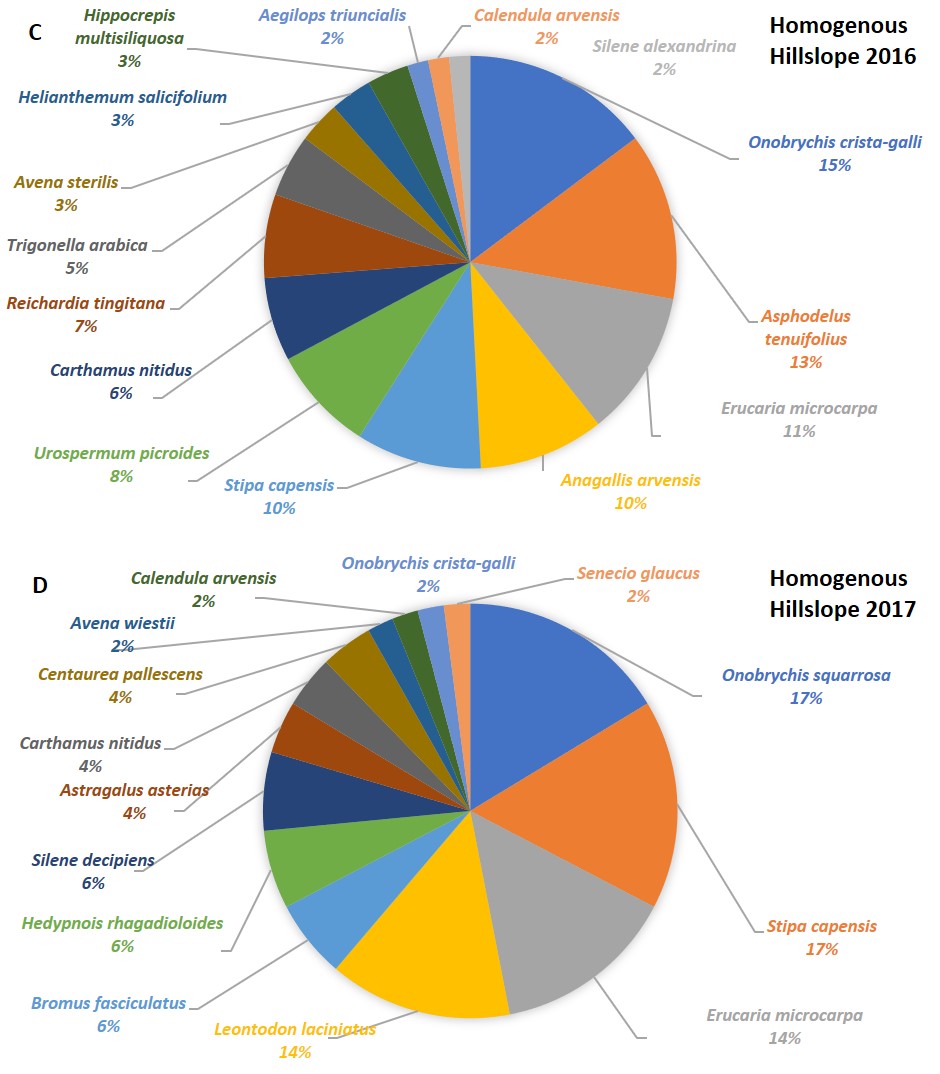

Supplement: Supplementary file 1 — Supplementary Information. [file 41598_2021_94698_MOESM1_ESM.docx]
